# Changes of Oral and Physical Functions in Older Japanese Who Participated in Keyboard Harmonica and Exercise Classes during COVID-19-Related Movement Restrictions

**DOI:** 10.3390/ijerph20043700

**Published:** 2023-02-19

**Authors:** Shinsuke Mizutani, Hideaki Matsuzaki, Kiyomi Iyota, Asuka Tani, Saori Oku, Hiroaki Tabuchi, Akiko Fujiwara, Shizuka Hase-Tamaru, Hiro Kishimoto, Kenji Narazaki

**Affiliations:** 1 Section of Geriatric Dentistry and Perioperative Medicine in Dentistry, Division of Maxillofacial Diagnostic and Surgical Sciences, Faculty of Dental Science, Kyushu University, 3-1-1 Maidashi, Higashi-ku, Fukuoka 812-8582, Japan; 2 OBT Research Center, Faculty of Dental Science, Kyushu University, 3-1-1 Maidashi, Higashi-ku, Fukuoka 812-8582, Japan; 3 Department of Rehabilitation Center, Fukuoka Mirai Hospital, 5-1 Kashiiteriha, Higashi-ku, Fukuoka 813-0017, Japan; 4 Department of Behavior and Health Sciences, Graduate School of Human–Environment Studies, Kyushu University, 744 Motooka, Nishi-ku, Fukuoka 819-0395, Japan; 5 Department of Life, Environment and Materials Science, Faculty of Engineering, Fukuoka Institute of Technology, 3-30-1 Wajiro-higashi, Higashi-ku, Fukuoka 811-0295, Japan; 6 Faculty of Arts and Science, Kyushu University, 744 Motooka, Nishi-ku, Fukuoka 819-0395, Japan; 7 Center for Liberal Arts, Fukuoka Institute of Technology, 3-30-1 Wajiro-higashi, Higashi-ku, Fukuoka 811-0295, Japan

**Keywords:** oral function, keyboard harmonica, physical activity, functional disability, older adults

## Abstract

Many older people have restricted activities or movements because of the coronavirus disease 2019 (COVID-19) pandemic, which causes concerns about secondary health problems. This study aimed to investigate how frailty-prevention activities implemented by local governments have changed the health of community-dwelling older people during the COVID-19 pandemic. In this observational study, the participants were 23 older Japanese people who took part in keyboard harmonica or exercise classes in 2021. Oral function examination and physical function tests were conducted at baseline and after 10 months of follow-up. In each class, the participants met 15 times and worked on assignments at home. The results showed that oral diadochokinesis/pa/, which represents lip dexterity, improved during 10 months (from 6.6 to 6.8 times/s, *p* < 0.046); however, grip strength (*p* < 0.005) and total skeletal muscle mass (*p* < 0.017) decreased in the keyboard harmonica group. In the exercise group, a statistically significant difference was found only in grip strength, which decreased (*p* < 0.003). The oral and physical functions of older people who participated in frailty-prevention activities implemented by local governments characteristically changed. Moreover, activity restrictions during the COVID-19 pandemic may have caused decreased grip strength.

## 1. Introduction

Since 2019, the world’s public health has been seriously threatened by the coronavirus disease 2019 (COVID-19) [1]. Many countries, including the United States, France, and China, have enacted lockdowns to prevent the spread of infection. In Japan, the first state of emergency was declared on 16 April 2020; since then, there have been a total of four declarations. Japan’s state of emergency is not legally binding but is a request for self-restraint. When the Japanese government declares a state of emergency, citizens are required to refrain from unnecessary and nonurgent social behaviors, including traveling, eating and drinking at a restaurant for extended periods, and socializing with groups of >5 people [2]. Therefore, many events were canceled, and public facilities were unavailable.

The COVID-19 pandemic has negatively affected physical activities [3], caused anxieties about walking ability and falls [4], and has influenced the mental health and social well-being [5] of older adults. For example, restrictions due to the COVID-19 pandemic have decreased the frequency of social participation and contact with family and friends among older Japanese people [6]. Moreover, Son et al. reported that social isolation influenced the muscle mass and strength of Japanese older women during the pandemic [7]. Therefore, given the movement and activity restrictions during the pandemic, many older adults faced an increased risk of secondary health issues [8,9].

Despite the need for appropriate management of frailty during the pandemic [10,11], the effect of the COVID-19 pandemic on the development of frailty is only beginning to emerge [12].

Previous studies have shown that frailty reduces an individual’s support resources due to limitations in mobility and social interactions, contributing to less social support among frail older adults than their non-frail peers [13]. It has been widely reported that older adults who possess less support are more likely to be depressed [14]. Furthermore, the International Conference of Frailty and Sarcopenia Research strongly recommended that all patients receive social support to improve or slow the progression of their frailty [15]. Therefore, social support is important for older people, and local governments in Japan have been working together to prevent frailty for successful aging. Despite the COVID-19 pandemic, the administration recently began to resume activities targeting residents. However, how such activities affect the health of community-dwelling older people is not yet known.

Our hypothesis was that the oral and physical functions of older people change via frailty prevention activities implemented by local governments. Therefore, this study aimed to investigate how frailty-prevention activities implemented by local governments and movement restrictions have changed the health of community-dwelling older people during the COVID-19 pandemic.

## 2. Materials and Methods

### 2.1. Participants and Study Design

**Figure 1:**
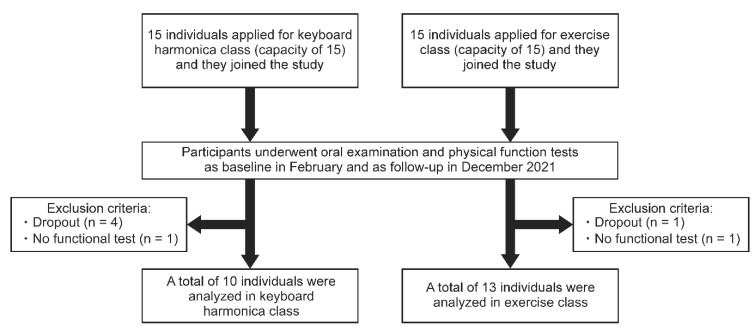
Flowchart of participant enrollment in the study.

This observational study was conducted in Koga City, Fukuoka Prefecture, in Japan between February and December 2021. The participants were 30 residents aged ≥60 years who enrolled in frailty-prevention classes, namely, keyboard harmonica and exercise classes (with 15 participants each), held by Koga City. The participants applied voluntarily after reading the city’s public relations magazine. Before each class started, they underwent oral examination and physical function tests in February 2021. Five participants dropped out over 10 months, and 25 received the same tests in December 2021. Those who could not complete the oral and physical function tests because of physical disabilities (n = 2) were excluded. Finally, the keyboard harmonica group and the exercise group were composed of 10 and 13 participants, respectively (Figure 1).

All participants were informed of the study and provided consent to participate before examinations. Furthermore, the participants were informed that the data obtained would not be used for other purposes or published and would not lead to any personal identification. They were free to withdraw from the study at any time. The Institutional Review Board of Fukuoka Institute of Technology (Approval No. hm01-21) and the Kyushu University Institutional Review Board (Approval No. 201912-1) approved the study following Helsinki Declaration guidelines.

### 2.2. Contents of Each Frailty-Prevention Class

Keyboard harmonica and exercise classes were part of the frailty-prevention class held by Koga City. The participants were recruited from among the residents, and classes were held annually for free.

In the keyboard harmonica class, the keyboard harmonica was loaned to everyone. The participants met twice a month at the city hall, received lectures, and practiced together. Specifically, the participants practiced the basic training, focusing on learning how to play and breathing exercises, and performed a set of selected songs for the class. The participants were also advised to practice the assigned works at home, although this was optional. In the exercise class, the participants gathered in the city hall twice a month, and all participants mainly performed strength training, stretching, and other exercises for the whole body. They were also instructed to use their spare time at home to perform optional exercises that can be easily carried out without the need for exercise devices. The contents of these classes were predetermined by the instructors, and the researchers were not involved.

The Japanese government declared a state of emergency due to the COVID-19 pandemic, and classes were not held from 12 May to 20 June 2021, and from 20 August to 30 September 2021. Therefore, both classes were held 15 times each with interruptions between February and December 2021.

### 2.3. Oral Function

Oral examinations were conducted by three well-trained dentists who did not know which class the participants were attending.

#### 2.3.1. Number of Remaining Teeth

Two dentists were responsible for this measurement. A penlight and dental tweezers were used for the examination, if they could confirm the tooth condition sufficiently. Teeth with residual roots not involved in occlusion and teeth with increased mobility (Miller’s classification 3) were excluded [16,17].

#### 2.3.2. Occlusal Force

The participants bit a pressure-indicating film (Dental Prescale II, GC Corporation, Tokyo, Japan) in the intercuspal position for 3 s. The occlusal force of the whole dentition was immediately measured using analyzing software (Bite Force Analyzer, GC Corporation, Tokyo, Japan) [18].

#### 2.3.3. Oral Diadochokinesis (ODK)

To determine ODK, tongue speed and smoothness and lip movements were comprehensively measured. The participants repeatedly pronounced the syllables /pa/,/ta/, and /ka/ in succession for 5 s for each syllable. The number of pronunciations per second was examined using a counter machine (Kenkokun Handy, Takei Scientific Instruments, Niigata, Japan). Generally, the count per second was less than six, and the tongue–lip motor function was considered decreased in older people [19].

#### 2.3.4. Tongue Strength

A dentist evaluated the maximal tongue pressure using a tongue pressure-measuring device (JMS Tongue Pressure Device; JMS Co., Ltd., Hiroshima, Japan). The participants raised their tongues and pressed the small balloon attached to the tip of a plastic probe between the roof of the mouth and the tongue with maximal spontaneous effort for 5 s. Measurements were taken three times with a 30-s rest interval, and the maximum value was adopted [17].

#### 2.3.5. Masticatory Performance

The examiners assessed the masticatory performance using a specially developed color-changeable gum (Masticatory Performance Evaluating Gum XYLITOL; Lotte Co., Ltd., Seoul, Republic of Korea), which changes color from green to red during chewing. The participants were instructed to chew this gum 60 times for 1 min. The change was measured visually on a scale of 1–10, with higher numbers indicating better masticatory performance and chewing power [20].

### 2.4. Physical Function

Physical function tests were conducted by staff who did not know which class the participants are attending.

#### 2.4.1. Grip Strength

Grip strength was measured using an electronic grip strength meter (Grip D TKK-5401, Takei Scientific Instruments Co. Ltd., Niigata, Japan). The second joint of the index finger was at a right angle to the grip meter. The participants were instructed to keep their feet apart, their arms naturally lowered, and their full grip on the meter. The measurements were repeated twice, alternating hands. The maximum value of four trials was adopted as an index of strength.

#### 2.4.2. Open-Eyed One-Leg Standing Time

Participants stood toward the wall at a distance of approximately 100 cm, comfortably lowered their arms with both eyes open, and raised either the right or left foot forward by approximately 5 cm. It was measured for up to 120 s to determine the duration the state could be kept. Right and left measurements were taken once, with the longest time being adopted as the representative value for one-leg standing time with eyes open [21].

#### 2.4.3. Five Times Sit-to-Stand Test

Participants sat on a chair and naturally opened their legs to approximately shoulder width, with their arms folded across the chest. After the examiner gave the start signal, the participants stands up from the chair and sits down again immediately after taking the upright standing position. The time required to repeat this motion 5 times was measured. This measurement was performed twice, and the quickest time was registered as the representative value [22].

#### 2.4.4. 5 m Gait Speed

Participants were instructed to walk for 11 m at the maximum walking speed possible, without running. The time was recorded between the 3rd and 8th m. Measurements were conducted twice, and the maximum value obtained was adopted as the indicator of declined walking speed [21].

#### 2.4.5. 3 m Timed up & Go Test (TUG)

Participants sat on chairs with their backs upright and their hands on their thighs. At the start signal, they stood up from the chair, walked to the landmark 3 m away without running, turned back, and sat back on the chair. The duration from when participants’ backs were separated from the chair to when they stood up and sat down again was measured. This measurement was performed twice, and the fastest time was adopted as the representative value [23].

### 2.5. Body Composition

Total skeletal muscle mass and total body fat was measured using a commercial multi-frequency body composition instrument (MC-780A-N, TANITA Co., Ltd., Tokyo, Japan). In addition, the body mass index (BMI) was calculated using weight and height.

### 2.6. Statistical Analysis

Given that all participants in both classes were included, no statistical sample size calculations were conducted. Fisher’s exact test for sex and the Mann–Whitney U test for the other characteristics were used to compare the keyboard harmonica and exercise groups at baseline. A two-way analysis of variance (time [baseline/follow-up] × group [keyboard harmonica group/exercise group]) was performed. In each group, the variables between baseline and follow-up were analyzed using the Wilcoxon signed-rank test. All statistical analyses were conducted using IBM SPSS Statistics for Windows version 28 (IBM Corp., Tokyo, Japan), with *p* < 0.05 indicating statistical significance.

## 3. Results

Table 1 shows the comparison of variables between the keyboard harmonica and exercise groups at baseline. No significant differences in physical functions and body composition, in addition to sex, age, and BMI, were found between the two groups. Although the tongue pressure in the keyboard harmonica group was lower than that in the exercise group (*p* < 0.002), no significant differences in other oral functions were found.

Table 2 shows the results of the two-way analysis of variance comparing variables between the keyboard harmonica and exercise groups at baseline and follow-up. The main effects of the interventions were observed in ODK/pa/(*p* = 0.040) and grip strength (*p* < 0.001), but the main effects of between groups were not. Interactions (intervention × between groups) were observed in ODK/pa/(*p* = 0.040) and five times sit-to-stand test (*p* = 0.010).

Table 3 shows the changes in variables at baseline and after follow-up in the keyboard harmonica group. ODK/pa/improved (*p* = 0.046), whereas the grip strength and total skeletal muscle mass decreased (*p* = 0.005 and 0.017, respectively). The sit-to-stand motion time and 3 m TUG time tended to extend, but no statistically significant differences were observed.

Table 4 shows the changes in variables at baseline and after follow-up in the exercise group. Grip strength decreased (*p* = 0.003) in the keyboard harmonica group. A tendency for reduced sit-to-stand motion time and 3 m TUG time was noted, but no statistically significant differences were found. On the contrary, oral functions tended to decrease, except for the occlusal force, but no statistically significant differences were noted.

## 4. Discussion

In this study, changes in oral and physical functions in older people who participated in keyboard harmonica or exercise classes were observed during the COVID-19 pandemic. We found that ODK/pa/, which represents lip dexterity, was improved in the keyboard harmonica group after 10 months. Moreover, grip strength and total skeletal muscle mass decreased. On the contrary, in the exercise group, a statistically significant difference was found only in grip strength, which indicated a decrease.

In the keyboard harmonica group, lip dexterity was further improved despite just preserving the function at baseline. A previous study reported that playing the harmonica influenced the performance of pursed-lip breathing, which is a recommended technique to help improve breathlessness in patients with COPD [24]. In our study, a keyboard harmonica instead of a harmonica was used, but the lip function was thought to improve because the mouthpiece was held in the mouth. Another intervention study using a keyboard harmonica revealed that the peak expiratory flow increased in patients with COPD [25]. Although we did not examine the respiratory function in consideration of the droplet transmission of COVID-19, further studies could reveal the effect of the keyboard harmonica on respiratory function in community-dwelling people. A previous study reported no statistically significant differences in ODK/ta/ and /ka/ between October 2019–February 2020 (before the first wave of the COVID-19 pandemic) and July–September 2020 (after the first wave of the COVID-19 pandemic) [7]. This was similar to our results; however, previous studies included only women, and our study had a small sample, so the results should be interpreted with caution.

Grip strength was significantly decreased in both groups, and the total skeletal muscle mass also decreased in the keyboard harmonica group. Although the exercise intervention in our study included a muscle-strengthening program, the grip strength would decrease even in the exercise class. Since physical activity levels in people older than 60 years are reduced during periods of restriction during the pandemic [26], several class interruptions might have contributed to the reduction in grip strength. Therefore, it may be important to investigate the changes over time, and we may need to focus on muscles to avoid “corona-frailty” [27], in addition to frailty prevention. For example, chair-based exercise as a physical activity can maintain balance, gait speed, and grip strength in older adults [28]. Furthermore, several studies have mentioned the associations between physical activity, mental health, quality of life, and well-being during the COVID-19 pandemic [29,30,31]. In addition to those factors, a further study with a larger sample size would need to consider exercise intensity [26], eating, and nutritional patterns [32].

Many studies have focused on social detachment among older adults during the COVID-19 pandemic. A previous study reported the association between social participation and muscle function in Japanese [7], and some reviews have indicated that loneliness and social isolation should be addressed [33,34]. In our study, frailty-prevention activities were held with the participants gathered together, and the city administration provided a place for social interaction. However, due to the declaration of a state of emergency, the classes were interrupted for a certain period, which might have made the older adults lonely. Although a recent review concluded that no intervention is effective in reducing social isolation and loneliness during the COVID-19 pandemic [35], the use of social network services and video calls may help older adults maintain social connection during the COVID-19 pandemic [12]. Therefore, as a new strategy, developing frailty-prevention measures using networks in addition to face-to-face meetings is considered essential.

This study has a few limitations. First, the sample size was small because the study investigated all participants in each class in 2021. Multi-year surveys may be required; however, the effect of the COVID-19 epidemic required attention. Second, since this study was conducted in a single city in Japan, whether the results can be generalized requires further discussion. Third, we did not investigate how the participants spent their time during the declaration of a state of emergency. Further studies could investigate social network usage, which may increase physical activities [36]. Finally, we did not examine the oral health-related quality of life (OHRQoL). According to a recent study, the COVID-19 pandemic and aggravated levels of depression, anxiety, and stress seem to be associated with lower OHRQoL [37].

This study revealed the differences in oral and physical functions between the two groups, but there was no proper control group. Thus, we could not conclude that these changes were the effect of interventions. Therefore, the results should be cautiously interpreted. However, even after the COVID-19 situation is under control, social gatherings and community activities will continue to be restricted for the time being. The results of this study may promote future research targeting frailty-prevention classes, which could lead to the provision of effective government services during the COVID-19 pandemic.

## 5. Conclusions

Oral and physical functions of older people who participated in frailty-prevention activities implemented by local governments exhibited characteristic changes; however, activity or movement restrictions due to the COVID-19 pandemic may decrease grip strength.

## Figures and Tables

**Table 1:** Comparison of variables between the keyboard harmonica and exercise groups at baseline.

Variables		Total (n = 23)	Keyboard Harmonica (n = 10)	Exercise (n = 13)	*p*-Value
Sex	Male	5 (22)	2 (20)	3 (23)	1.000 *
	Female	18 (78)	8 (80)	10 (77)	
Age		72 (68.0, 74.0)	72 (69.0, 75.0)	70 (65.5, 73.0)	0.284 ^†^
Class participation (times)		14.0 (14.0, 15.0)	14.5 (14.0, 15.0)	14.0 (13.0, 15.0)	0.446 ^†^
Number of remaining teeth		25.0 (22.0, 28.0)	25.0 (19.8, 25.8)	28.0 (24.0, 28.0)	0.101 ^†^
Occlusal force (N)		771 (617, 1117)	720 (544, 870)	884 (583, 1222)	0.208 ^†^
ODK/pa/(times/s)		6.6 (6.2, 7.0)	6.6 (5.5, 7.0)	6.6 (6.2, 7.1)	0.648 ^†^
ODK/ta/(times/s)		6.8 (6.4, 7.4)	6.8 (5.7, 7.3)	7.0 (6.4, 7.5)	0.284 ^†^
ODK/ka/(times/s)		6.4 (6.2, 6.6)	6.6 (5.6, 6.7)	6.2 (6.2, 6.7)	0.738 ^†^
Tongue pressure (kPa)		35.3 (31.7, 40.0)	31.6 (23.9, 34.7)	37.3 (35.1, 43.0)	0.002 ^†^
Masticatory performance		8.0 (8.0, 9.0)	8.0 (8.0, 8.3)	8.0 (8.0, 9.0)	0.483 ^†^
Grip strength (kg)		28.0 (23.5, 32.5)	27.0 (23.0, 34.6)	29.0 (24.5, 32.5)	0.648 ^†^
Open-eyed one-leg standing time (s)		120 (58, 120)	107 (62, 120)	120 (42, 120)	0.784 ^†^
Five times sit-to-stand test (s)		6.97 (5.85, 9.22)	6.38 (5.46, 8.30)	7.29 (6.02, 9.36)	0.284 ^†^
5 m gait speed (s)		3.26 (2.79, 3.82)	3.27 (2.79, 3.72)	3.17 (2.80, 3.84)	0.987 ^†^
3 m TUG (s)		5.40 (4.60, 6.95)	5.28 (4.73, 6.87)	5.40 (4.60, 7.01)	0.976 ^†^
Total skeletal muscle mass (kg)		35.3 (33.4, 39.0)	35.6 (32.6, 40.4)	35.2 (33.0, 42.0)	0.948 ^†^
Total body fat (kg)		15.0 (11.2, 17.1)	14.8 (10.7, 18.2)	15.1 (11.5, 17.0)	0.744 ^†^
BMI		21.4 (20.5, 23.2)	21.2 (20.1, 25.3)	21.4 (19.9, 22.4)	0.784 ^†^

Data are shown as number (%) or median (25th and 75th percentiles). * Fisher’s exact test; ^†^ Mann–Whitney U test. BMI, body mass index; ODK, oral diadochokinesis; 3 m TUG, 3 m timed up and go.

**Table 2:** Comparison between the keyboard harmonica and exercise groups at baseline and follow-up.

	Keyboard Harmonica (n = 10)		Exercise (n = 13)		Main Effect		Interactions (Time × Between Groups)
					Time	Between Groups	
Variables	Baseline	Follow-Up	Baseline	Follow-Up	*p*-Value *	*p*-Value *	*p*-Value *
Occlusal force (N)	696 ± 293	643 ± 343	899 ± 319	910 ± 318	0.680	0.072	0.528
ODK/pa/(times/s)	6.3 ± 1.1	6.8 ± 0.4	6.7 ± 0.4	6.7 ± 0.5	0.040	0.597	0.040
ODK/ta/(times/s)	6.5 ± 0.9	6.7 ± 0.6	7.0 ± 0.6	6.7 ± 0.7	0.633	0.351	0.074
ODK/ka/(times/s)	6.2 ± 0.7	6.2 ± 0.6	6.5 ± 0.5	6.3 ± 0.5	0.527	0.385	0.320
Tongue pressure (kPa)	30.9 ± 6.7	32.1 ± 6.6	39.2 ± 5.2	35.9 ± 5.1	0.441	0.008	0.114
Masticatory performance	8.1 ± 0.6	7.8 ± 1.2	8.2 ± 0.8	8.2 ± 0.6	0.394	0.368	0.394
Grip strength (kg)	29.7 ± 8.6	27.3 ± 7.9	31.1 ± 9.8	28.9 ± 9.5	<0.001	0.694	0.890
Open-eyed one-leg standing time (s)	96 ± 28	101 ± 28	85 ± 47	96 ± 40	0.134	0.572	0.544
Five times sit-to-stand test (s)	6.83 ± 1.70	7.69 ± 2.24	7.59 ± 1.69	6.79 ± 1.71	0.915	0.915	0.010
5 m gait speed (s)	3.40 ± 0.76	3.85 ± 1.59	3.33 ± 0.56	3.26 ± 0.27	0.465	0.241	0.321
3 m TUG (s)	5.72 ± 1.14	6.00 ± 0.86	5.77 ± 1.37	5.76 ± 1.11	0.280	0.844	0.230
Total skeletal muscle mass (kg)	37.1 ± 6.6	36.6 ± 6.2	37.3 ± 6.4	37.3 ± 6.7	0.226	0.877	0.201
Total body fat (kg)	15.2 ± 5.9	15.7 ± 5.9	14.5 ± 4.7	13.7 ± 3.8	0.706	0.525	0.115

Data are shown as mean ± standard deviation. * Two-way ANOVA (time × between groups); ODK, oral diadochokinesis; 3 m TUG, 3 m timed up and go.

**Table 3:** Within-group comparison of each endpoint at baseline and follow-up in the keyboard harmonica group (n = 10).

Variables	Baseline	Follow-Up	*p*-Value *
BMI	21.2 (20.1, 25.3)	21.2 (19.4, 24.9)	0.798
Occlusal force (N)	720 (544, 870)	630 (327, 969)	0.799
ODK/pa/(times/s)	6.6 (5.5, 7.0)	6.8 (6.6, 7.1)	0.046
ODK/ta/(times/s)	6.8 (5.7, 7.3)	6.6 (6.6, 7.1)	0.491
ODK/ka/(times/s)	6.6 (5.6, 6.7)	6.2 (5.8, 6.7)	0.856
Tongue pressure (kPa)	31.6 (23.9, 34.7)	30.8 (27.4, 36.6)	0.374
Masticatory performance	8.0 (8.0, 8.3)	8.0 (7.0, 9.0)	0.414
Grip strength (kg)	27.0 (23.0, 34.6)	24.5 (21.4, 33.2)	0.005
Open-eyed one-leg standing time (s)	107 (62, 120)	120 (69, 120)	0.500
Five times sit-to-stand test (s)	6.38 (5.46, 8.30)	7.26 (5.79, 10.22)	0.139
5 m gait speed (s)	3.27 (2.79, 3.72)	3.25 (2.76, 4.52)	0.959
3 m TUG (s)	5.28 (4.73, 6.87)	5.60 (5.26, 6.86)	0.203
Total skeletal muscle mass (kg)	35.6 (32.6, 40.4)	35.0 (32.4, 39.5)	0.017
Total body fat (kg)	14.8 (10.7, 18.2)	14.8 (11.5, 19.1)	0.214

Data are shown as median (25th and 75th percentiles). * Wilcoxon signed-rank test; BMI, body mass index; ODK, oral diadochokinesis; 3 m TUG, 3 m timed up and go.

**Table 4:** Within-group comparison of each endpoint at baseline and follow-up in the exercise group (n = 13).

Variables	Baseline	Follow-Up	*p*-Value *
BMI	21.4 (19.9, 22.4)	21.4 (20.0, 22.2)	0.123
Occlusal force (N)	884 (583, 1222)	909 (657, 1086)	0.753
ODK/pa/(times/s)	6.6 (6.2, 7.1)	6.6 (6.1, 7.3)	0.713
ODK/ta/(times/s)	7.0 (6.4, 7.5)	6.4 (6.2, 7.4)	0.070
ODK/ka/(times/s)	6.2 (6.2, 6.7)	6.2 (5.9, 6.7)	0.141
Tongue pressure (kPa)	37.3 (35.1, 43.0)	36.2 (31.6, 39.0)	0.055
Masticatory performance	8.0 (8.0, 9.0)	8.0 (8.0, 9.0)	1.000
Grip strength (kg)	29.0 (24.5, 32.5)	24.7 (23.5, 32.1)	0.003
Open-eyed one-leg standing time (s)	120 (42, 120)	120 (59, 120)	0.173
Five times sit-to-stand test (s)	7.29 (6.02, 9.36)	6.29 (5.41, 8.13)	0.100
5 m gait speed (s)	3.17 (2.80, 3.84)	3.26 (3.07, 3.51)	0.700
3 m TUG (s)	5.40 (4.60, 7.01)	5.22 (4.96, 6.97)	0.753
Total skeletal muscle mass (kg)	35.2 (33.0, 42.0)	34.9 (32.4, 42.8)	0.807
Total body fat (kg)	15.1 (11.5, 17.0)	14.2 (10.8, 17.2)	0.308

Data are shown as median (25th percentiles, 75th percentiles). * Wilcoxon signed-rank test; BMI, body mass index; ODK, oral diadochokinesis; 3 m TUG, 3 m Timed up and go.

## Data Availability

The data that support the findings of this study are possibly available on reasonable request from the corresponding author, K.N. The data are not publicly available due to restrictions, e.g., their containing information that could compromise the privacy of research participants.
